# Caregivers' perceived treatment failure in home-based management of fever among Ugandan children aged less than five years

**DOI:** 10.1186/1475-2875-5-124

**Published:** 2006-12-15

**Authors:** Mugagga Malimbo, Erieza Mugisha, Fred Kato, Charles Karamagi, Ambrose O Talisuna

**Affiliations:** 1 Ministry Health, Epidemiological Surveillance Division, Po Box 7272, Kampala, Uganda; 2 Rakai District Administration, Health Department, Uganda; 3 Clinical Epidemiology Unit, Makerere University, Kampala, Uganda

## Abstract

**Background:**

Home-based management of fever (HBMF) could improve prompt access to antimalarial medicines for African children. However, the perception of treatment failure by caregivers has not been assessed.

**Methods:**

Caregiver's perceived treatment outcome in HBMF and in alternative sources of fever treatment was assessed in a rural Ugandan setting using nine hundred and seventy eight (978) caregivers of children between two and 59 months of age, who had reported fever within two weeks prior to the study.

**Results:**

Lower caregivers' perceived treatment failure (15% and 23%) was observed in the formal health facilities and in HBMF, compared to private clinics (38%), drug shops (55%) or among those who used herbs (56%). Under HBMF, starting treatment within 24 hours of symptoms onset and taking treatment for the recommended three days duration was associated with a lower perceived treatment failure. Conversely, vomiting, convulsions and any illness in the month prior to the fever episode was associated with a higher perceived treatment failure.

**Conclusion:**

In this medium malaria transmission setting, caregiver's perceived treatment outcome was better in HBMF compared to alternative informal sources of treatment.

## Background

Malaria is one of the leading causes of ill health and deaths in Uganda, accounting for 46% of the illness in children, 20–40% of out-patient visit at health facilities, 25% of hospital admissions, 14% of in-patient deaths and 20–23% of – infant and childhood mortality (Uganda Ministry of Health,, surveillance reports, 2001). High malaria transmission intensity, limited access to adequate treatment, increasing parasite resistance to affordable and safe medicines (chloroquine, sulphadoxine-pyrimethamine and amodiaquine);, delayed seeking of care and inappropriate treatment at home or within communities, are some of the causes for this pervasive situation. Based on the observation that the majority of fevers in Africa are due to malaria [1], and evidence demonstrating that home management of malaria (HMM) reduces childhood morbidity and mortality [2-4], some countries have adopted the World Health Organization home-based management of fever (HBMF) strategy in an effort to improve prompt access to treatment. In Uganda, village volunteers, called community drug distributors (CDD) provide free pre-packaged antimalarial medicines (HOMAPAK) to caregivers of febrile children. Caregivers visit the CDD when they have a sick child and seek further treatment in case of perceived treatment failure. The HBMF strategy has enabled over 52% of the febrile children in target areas to access treatment within 24 hours of fever onset, and data from CDDs demonstrate a high recovery (79% – 99%) [5]. Despite such a high recovery, the out-patient department (OPD) attendance at health facilities in the districts implementing HBMF has increased since the inception of HBMF, yet a decline had been anticipated. Several factors could be responsible including (i) increased availability of drugs (fewer stock-outs) resulting in increased overall health facility contacts per person per year, (ii) increased access to health services through the opening up of new health centres; (iii) HBMF serving the hard-to-reach population, that previously never attended formal health care, consequently there is little or no effect on OPD attendance; (iv) surveillance bias due to improved completeness of reporting, and finally (v) increased parasite resistance to chloroquine (CQ) + sulphadoxine-pyrimethamine (SP), the present regimen in HBMF. Indeed, the high parasite resistance to CQ + SP has led to concerns that the effectiveness of HBMF was very low, [6-10], probably as low as 4 to 6% [11].

In 2004, faced with widespread parasite resistance, Uganda has changed the antimalarial drug policy for uncomplicated malaria to artemether-lumefantrine (AL), (Coartem^®^). However, several concerns have been raised with respect to the use of artemisinin derivative-based combination therapy (ACT) in HBMF because of little experience, in Africa, with ACTs outside clinical trials [12,13]. The main concerns include: the feasibility and acceptability of ACTs in HBMF, the adherence by caregivers and the dosing schemes too complex for CDDs, the risk of increasing the spread of drug resistance, the high cost of ACT, the potential for wastage and the uncertainties in the supply of ACTs. However, a contrary view is that ACT is the only available " bullet" in the face of increasing drug resistance and that it would not be ethically and morally right to continue using cheap drugs with sub-optimal efficacy for poor people [14].

The prevalence of caregivers' perceived treatment failure (CP-TF) in the formal and informal sectors, as well as in HBMF, the factors associated with treatment outcome and the corrective actions taken by caregivers in case of perceived treatment failure were assessed in a rural Ugandan setting.

## Methodology

### Study setting

The study was conducted in two counties (Kakuuto and Kooki) in Rakai district, located approximately 200 kilometers south of the capital city, Kampala. Rakai district has meso-endemic malaria and is composed of four counties which are predominantly rural (the majority of the residents practice subsistence farming). According to the 2002 population census, the total population of Rakai district was 470,365 of whom 20% were children less than five years of age. The study counties were composed of 12 sub counties, 43 parishes and 273 villages. The implementation of HBMF commenced in 2002.

### Study design

A community-based cross-sectional study was conducted using quantitative and qualitative data collection techniques.

### Quantitative data collection

Consenting caregivers of children between two and 59 months of age, who reside in the study counties and had a child with fever in the two weeks prior to the study were interviewed. Caregivers of children who were on medication and those who could not provide the required information or refused to consent were excluded. A two-stage cluster sampling method was used in which the parish level was taken as a cluster. At the first stage, a cumulative list of the households in the study parishes was prepared and used to select the parishes (clusters) by probability proportional to size (PPS). For the selection of households, a local guide facilitated the location of a central place in the parish, and the direction to follow in the selection of households was determined by randomly spinning a bottle. The first household with a child less than five years old, in the direction pre-determined through the bottle spin was selected and the immediate next household with a child of the target age was repetitively selected until 40 caregivers of children had been selected in a parish. Data for several variables from the caregivers' were collected including; socio-demographic characteristics (age, sex, religion, place of residence, tribe/ethnic group and the highest education level achieved). In addition, child related data were also collected including: the age, the relationship of the child to the caregiver and the number of children less than five years old in the household. Further, data on accessibility to health services including; availability of the HBMF CDD in the parish, distance to nearest formal government/NGO health facility and presence of a private clinic/drug shop in the village were also collected. Treatment seeking for malaria/fever episode or the health seeking behavior of the respondents was assessed by obtaining information on the time lag between onset of fever and seeking treatment; the source of initial treatment; and the type and place of additional treatment received. Adherence to treatment and other drug related factors was assessed by collecting information on the dose administered; the dosing duration and whether the drugs were shared with another person.

### Qualitative data collection

Twelve key informant interviews and four focus group discussions (FGDs) were conducted to provide explanatory information. The key informants included CDDs, the staff in charge of health facilities and district health management team officials. The FGDs were grouped by area of residence. A social scientist moderated the discussions and one of the other investigators recorded the notes.

### Sample size estimation

The sample size estimates were derived as suggested previously [15,16] based on the following assumptions; a tolerable sampling error of 5%, fever prevalence for children aged less than five years old in HBMF intervention areas of 47% [5]; and a design effect of two. The number of respondents per cluster was set at 40 for convenience and the required number of clusters was 19.5 (rounded off to 20). A pre-study sample size of 800 respondents was derived to cater for non responses and missing data.

### Data management and statistical analysis

Data were entered into EPIINFO 6.04 (CDC, Atlanta GA), cleaned and analysed in SPSS 12.0 and Stata version 9.0 (Stata Corp, Texas USA). The prevalence of caregivers perceived treatment failure was defined as the proportion of children who were treated and failed to recover (subjectively reported by the caregivers) out of the total number treated. The factors associated with treatment failure were determined by carrying out bi-variate and multivariate analysis. For the bi-variate analysis, associations between independent variables and the outcome variable (CP-TF) were assessed. Continuous independent variables were categorized and their association with the outcome variable was established using Chi-square tests or fishers exact test (for tables where cell values were less than five). The measure of association for categorical variables was the odds ratios (OR) with their 95% confidence intervals and P values less than 0.05 were considered significant. In the multivariate analysis, statistically significant factors at bi-variate analysis were chosen. Confounding and interaction between the various factors was assessed using logistic regression.

Qualitative data from the FGDs and KI interviews, conducted in the local language (Luganda) were translated and transcribed into English, coded and thereafter analysed and separated into the emerging themes.

### Quality control

Carefully chosen research assistants were trained on how to conduct interviews and to make good observations. The research instruments were translated into the local language (Luganda). To improve validity, the semi-structured questionnaire was pre-tested before data collection. An operation manual was prepared and regular meetings with the research assistants were conducted. Completed questionnaires were cross-checked in the field to ensure completeness and proper recording of the data.

### Ethical considerations

Ethical clearance to conduct the research was sought from the Makerere University Clinical Epidemiology Unit, Faculty of Medicine Research and Ethics Committee, and the Rakai District Local Government. A written informed consent was sought from all the caregivers and permission to be admitted into the village was sought from the village local councils.

## Results

### Social demographic characteristics

A total of 798 caregivers (coverage = 99%) were interviewed on the treatment actions taken in response to fever episodes occurring in the children less than five years of age in the two weeks prior to the study. The median age of the caregivers was 29 (range: 15–87) years and the mean age was 31 (SD = 10.33) years. The median age for the children was 25 (range: 2–59) months and the mean age was 27 (SD = 15.7) months. Females comprised 88% (704/798) of the caregivers and approximately 40% (320/798) of the caregivers had attained an educational level of upper primary (Primary 5 to 7). Less than 2% (12/798) of the respondents had achieved an education above secondary level. The majority of the caregivers (82%, 653/798) were married and over 90% (724/798) were subsistence farmers. The total number of household members varied from 2 to16 (mean = 6 people/household) and the number of children below five years of age in the households varied from 1 to 6 (mean = 2) years. Caregivers who were mothers of the children were 79% (628/798), while fathers were only 10% (77/798). Grandparents and other relatives were caregivers for 8% (66/798) and 3% (27/798) of the children respectively. Almost 75% (594/798) of the children lived in households headed by fathers. Grandparents and mothers headed 14% (108/798) and 9% (74/798) of the households respectively, while other relatives headed 3% (22/798) of the households.

### Access to Health Services

Over 95% (777/798) of the caregivers lived within a five kilometer radius from a government or a private not-for-profit (PNFP) health facility. The mean distance to the nearest government or PNFP health facility was about two kilometers. The PNFP/NGO health units that charge user fees were the nearest formal health facilities for approximately 25% (201/798) of the caregivers. Where a CDD existed, over 90% (503/552) of caregivers lived a kilometer or less from them. Surprisingly, over 30% (246/798) of the caregivers were not aware of the existence of an active CDD for HBMF in their village. The leading source of information on health (76%, 603/798) was the radio, followed by health workers at 42% (335//798).

### Child related factors

**Figure 1:**
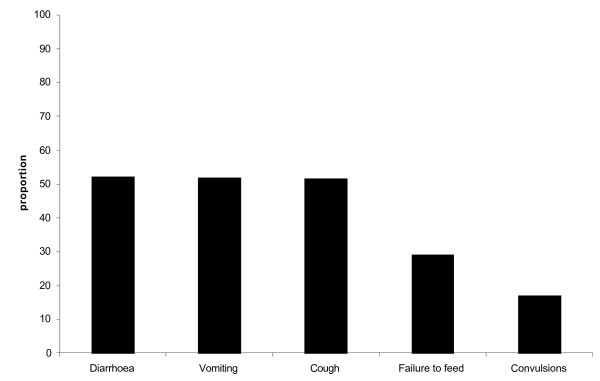
Common symptoms for children with fever in the two weeks prior to the study, Rakai, Uganda, 2006.

The most common symptoms accompanying fever among the children in descending order were: diarrhea, vomiting, cough, failure to feed, and headache (Figure 1). Over two thirds (532/798) of the children had an immunization card showing the immunization status, while another 15% (118/798) of the children did not have a card but reported that they had received the recommended immunization. About 19% (148/798) of the children had not received the recommended immunization for their age.

### Treatment seeking for a fever episode

**Figure 2:**
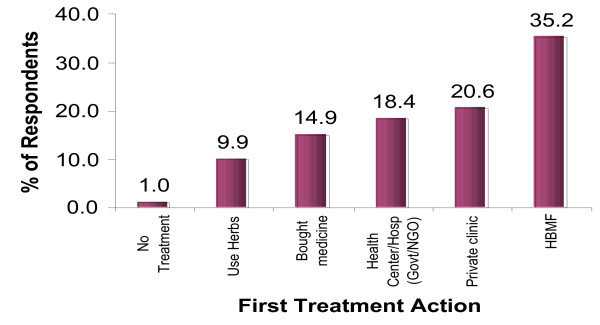
Caregivers' perceived treatment failure, according to first action taken for children with fever.

Administration of HOMAPAK obtained from the CDD was the most common first treatment action (Figure 2). Surprisingly, caregivers with a higher education level tended to use HBMF more (p = 0.009) (Table 1). Neither the marital status nor occupation of the respondent was associated with using HBMF as the first treatment action. The factors associated with using HBMF/HOMAPAK were: distance to health facility (p = 0.004); distance to the CDD (p < 0.001); having health workers as a major source of health information (p = 0.016) or politicians/local leaders as a major source of health information (p = 0.035).

**Table 1:** Demographic characteristics of 798 caregivers by first treatment action taken, Rakai Uganda, 2006

	**Total**	**No Treatment%**	**Use Herbs%**	**Bought medicine%**	**Health Centre/Hosp (Govt/NGO)%**	**Private clinic%**	**HBMF%**
**Education level**							
Never been to School	138	0.0	10.1	17.4	26.1	21.7	24.6
Primary	226	1.5	11.0	13.4	17.4	20.3	36.4
Post primary	320	0.0	4.4	19.3	4.0	20.2	42.1

**Religion**							
Catholic	439	1.4	11.4	14.4	17.5	16.4	39.0
Protestants	201	0.5	8.0	14.4	20.4	27.9	28.9
Moslems	93	0.0	5.4	12.9	21.5	21.5	38.7
Other Christians	35	2.9	14.3	22.9	14.3	20.0	25.7
Others	30	0.0	10.0	23.3	13.3	30.0	23.3

**Occupation**							
Peasant Farmer	724	0.8	10.4	14.4	19.2	20.4	34.8
Private Business	39	2.6	7.7	17.9	7.7	23.1	41.0
Civil Servant	17	0.0	5.9	11.8	23.5	11.8	47.1
Others	4	5.6	0.0	33.3	5.6	27.8	27.8

**Marital Status**							
Married/living with spouse	653	0.8	9.6	14.7	19.6	21.4	33.8
Widowed	54	0.0	9.3	7.4	16.7	20.4	46.3
Divorced/Separated	45	2.2	13.3	22.2	11.1	15.6	35.6
Single/Never married	46	4.3	10.9	19.6	10.9	13.0	41.3

### Caregivers' perceptions on effectiveness of HBMF

The caregivers who had an active CDD in their village were asked to comment on their satisfaction with HBMF in fever management and 64% of them reported that they were satisfied with the strategy. Several reasons for their satisfaction were cited including; free provision of drugs; easy accessibility to the CDD who readily explain to the caregivers. Among the caregivers who were not satisfied with HBMF, the most common reason cited was using HOMAPAK previously without recovery; lack of confidence in a non-medical person prescribing the treatment; occurrence of stock-outs; and long distance from the CDD.

### Prevalence of treatment failure according to source of treatment

The overall prevalence of treatment failure was 38% (95% CI: 34.1–42.7). Of the 281 children treated under HBMF as the first treatment action for fever, 23% (95% CI: 18.0–28.1) did not recover. However, among children treated in government and NGO health facilities the prevalence of treatment failure was 15% (95% CI: 9.6–21.8), while in the private for profit sources (private clinics and drug shops) the prevalence of treatment failure was 45% (95% CI 39.3 – 51.2) (Table 2). The presence of vomiting, failure to feed, convulsions, extreme weakness and difficulty in breathing were all associated with a higher likelihood of perceived treatment failure (Table 3). The odds of treatment failures were four times higher among children who started treatment on the second day after the commencement of symptoms compared to those who started treatment within 24 hours of recognizing the symptoms. Further, the odds of failure increased to over nine times more among those who started treatment after 72 hours (Table 4). There was an increased CP-TF (OR = 2.28, 95% CI 1.30 – 4.02) among children who had been ill within a month of the last episode of fever. Those who took medication for only one day were three and a half times more likely to experience treatment failure than those who completed treatment in the recommended duration, while those who terminated treatment on second day had a more than four-fold increase in CP-TF compared to those who completed treatment (Table 4).

**Table 2:** Caregivers' perceived treatment failure by first treatment action, Rakai District, Uganda, 2006

**Treatment Action**	**N**	**CP-TF% (n)**	**95% CI**
Health Centre/Hosp (Government/NGO)	147	15.0 (22)	9.6 – 21.8
HBMF	281	22.8 (64)	18.0 – 28.1
Private clinic	164	38.4 (63)	30.9 – 46.3
Drug shop	119	54.6 (65)	45.2 – 63.8
Use Herbs	79	55.7 (44)	44.1 – 66.9
**Overall**	**798**	**32.8 (262)**	**29.6 – 36.2**

**Table 3:** Caregivers' perceived treatment failure and reported symptoms among 281 under-five year old children treated under HBMF, Rakai District, Uganda, 2006

**Symptom**	N*	**CP-TF% (n)**	**OR (95% CI)**	**P-value**
Failure to eat, drink or Breast feed	85	14.1 (12)	0.46 (0.23–0.91)	0.02
Headache	42	19.0 (8)	0.77 (0.33–1.76)	0.5
Diarrhea	129	20.9 (27)	0.82 (0.47–1.46)	0.5
Cough	136	25.0 (34)	1.29 (0.73–2.23)	0.4
Extreme weakness	28	39.3 (11)	2.44 (1.08–5.53)	0.03
Difficult breathing	20	45.0 (9)	3.06 (1.21–7.77)	0.01
Vomiting	124	33.9 (42)	3.14 (1.75–5.64)	0.000
Convulsions	51	58.8 (30)	8.24 (4.23–16.0)	0.000

*Total numbers are more than 281 because most children had multiple symptoms

**Table 4:** Perceived treatment failure, time lag to receiving treatment after symptoms onset and adherence to duration of treatment among under-five children treated under HBMF, Rakai District, Uganda 2006

**Period to seeking treatment after onset of symptoms**	**N = 281 n (%)**	**CP-TF% (n)**	**OR (95% CI)**	**p-value**
Same day (within 24 hours)	162 (57.7)	10.5 (17)	Reference	
Second day	79 (28.1)	32.9 (26)	4.18 (2.10–8.32)	0.000
Three or more days	40 (14.2)	52.5 (21)	9.43 (4.24–20.95)	0.000

**Duration of taking HOMAPAK**				

Three days and more	235 (83.6)	17.9 (42)	Reference	
One day	7 (2.5)	42.9 (3)	3.45 (0.74–5.98)	0.094
Two days	38 (13.5)	50.0 (19)	4.60 (2.24–9.42)	0.000

In a multivariate analysis, the factors independently associated with a lower treatment failure under HBMF were: timeliness from onset of symptoms to commencement of treatment (OR = 0.10, 95% CI: 0.04–0.26) and completion of recommended treatment duration (OR = 0.3, 95% CI: 0.13–0.69). The factors that increased CP-TF included: vomiting (OR = 5.02, 95% CI: 2.31–10.92), convulsions (OR = 10.51 95% CI: 4.46–24.77) and history of illness within a month prior to the onset of the fever episode treated under HBMF (OR = 2.46, 95% CI: 1.18–5.13) (Table 5).

**Table 5:** Factors independently associated with perceived treatment failure among 281 under-five children treated under HBMF, Rakai District, 2006

**Factor**	**N = 281**	**CP-TF n (%)**	**Adjusted OR 95% CI)**
Treatment within 24 Hrs	162	10.5 (17)	0.10 (0.04–0.26)
Adherence to recommended duration of treatment	235	17.9 42	0.30 (0.13–0.69)
Treatment within 25–48 hours	79	32.9 (26)	0.32 (0.12–0.83)
Ill in the previous month	106	32.1 (34)	2.46 (1.18–5.13)
Vomiting	124	33.9 (42)	5.02 (2.31–10.92)
Convulsions	51	58.8 (30)	10.51 (4.46–24.77)

Of the 281 children treated under HBMF, 77.2% (217/281) did not need to seek further treatment because they had fully recovered, 11% (31/281) of them sought further treatment from government or NGO health facility while 7% (20/281) went to private clinics. In less than 1% (2/281), the caregivers used HOMAPAK again after the children had failed to improve. Considering only those who did not improve under HBMF as the first treatment, 48% (31/64) sought additional care from the government or NGO health facilities, while 31% (20/64) went to private clinics (Table 6).

**Table 6:** Treatment actions taken following perceived treatment failure among 281 under-five children who used HBMF as the first treatment action, Rakai District, 2006

**Treatment Action**	**N = 281 n (%)**	**95% CI**
Required no more treatment	217 (77.2)	71.9–82.0
No treatment	3 (1.1)	0.2–3.1
Use herbs	3 (1.1)	0.2–3.1
Traditional healer	2 (0.7)	0.1–2.5
Bought medicine	3 (1.1)	0.2–3.1
Health centre/Hosp government/NGO)	31 (11.0)	7.6–15.3
Private clinic	20 (7.1)	4.4–10.8
HBMF	2 (0.7)	0.1–2.5

### Qualitative assessments

#### Uptake of HBMF

The key informants and focus group discussions demonstrated that the most commonly used source of treatment for fever in the study area was HBMF. The following statements describe some of the experiences:

"*It is now easy... when a child gets fever you go to the drug distributor. It is a big relief... you don't need to have money and the distributors are near to our homes (FGD, Lwanda Parish)*"

Key informants attributed the uptake of HBMF to high sensitization of the communities about the strategy, which they reported had helped to reduce congestion in health facilities.

"You can't imagine how many children we used to attend to... nowadays we mainly get severe cases. If children could be started on treatment early, even the severe cases would be very few". (Male Key Informant, health unit in-charge)

Easy access to drugs at all times of the day and from people well known to the community was reported to be a big attraction to the HBMF strategy.

"*I grew up in this village... everybody knows me, that is why I was selected to be a drug distributor. People like this treatment because it works... we were taught to serve our people and we try to do our best. That is why they have confidence in me because when the illness is severe I refer them to the Health Centre unlike the traditional healers (Female Key Informant, Community Drug Distributor)*.

#### Reduced effectiveness of the HBMF

Some focus groups, however, indicated that the HBMF program is no longer as active as before. "*When this program started everything was ok... these days you can't be sure that the drugs will be there, therefore you resort to private clinics which are nearer than the health centre and they always have drugs (FGD, Kakuuto parish)*. In addition, there was a perception of increasing treatment failure among children treated under HBMF from both key informants and focus groups.

"*What we are talking about is chloroquine and Fansidar (sulphadoxine/pyrimethamine)... we know their fate and that is why the government is changing the anti-malarial policy. Surprisingly we still get many children who improve on HOMAPAK although the number of those who fail to improve appears to be on the increase" (Key informant, health unit in-charge)*.

The focus groups also reported a declining effectiveness of HBMF strategy. *"May be when you use these drugs for long they get weak... some fevers are too strong for these oral drugs... that is why sometimes children fail to improve. We have also discovered that when a child takes HOMAPAK without Panadol (Paracetamol), the chances of improving are few" (FGD, Lwensinga parish)*.

### Sustainability

There is a perception that in future the programme may not be sustainable unless efforts are taken to improve the incentive scheme for CDDs.

*" The government provides the drugs and trains the CDD... it remains a challenge to determine how much more the government can add to facilitate the program. Our hope is that community members should assist the government to facilitate the CDD. (Key Informant, CDD)*.

*We bear a lot of burden for this program... we collect the drugs from health centres, sometimes we wake up at night to give the treatment, we sometimes provide transport for referring severely ill children and we incur a lot more costs, with no refund from government. Some people think we are paid, so they put pressure on us. I am about to give up if things remain like this (Female Key Informant, CDD)*.

The focus groups also demonstrated that although the program is presently functional, its future is not clear.

The CDDs try... but I don't think they will go on for ever without some form of facilitation. Even in our tradition, herbalists get some payment (ekikuba nsiko), how do you expect them (CDD) to serve us well without any payment? (FGD, Kakuuto)

## Discussion

A client-focused approach was used to assess treatment outcome for different sources of fever treatment including HBMF and has provided seminal and important information to improve HBMF implementation in Uganda and in other malaria endemic countries. The caregivers' perceived treatment failure under HBMF was rather high. Nevertheless, the treatment outcome under HBMF was better than that for private clinics and drug shops or herbalists, suggesting that HBMF still offers better treatment outcomes compared to alternative informal treatment actions accessible to rural communities. At the time of the present study, CQ plus SP was the first line regimen in the formal health facilities and in HBMF. Therefore, a possible explanation for the high perceived treatment failure in HBMF is likely to be the high resistance to CQ plus SP which eventually led to a change of the anti-malarial treatment policy [6-10]. This study from a caregivers' perspective, therefore, supports earlier evidence from clinical trials that the present regimen (CQ+SP) in the Ugandan HBMF has sub-optimal treatment efficacy.

Home-based management of fever was the major treatment action taken by caregivers in response to fever in children aged less than five years. Considering that only 35% and 18% of the children were treated under HBMF and at government/NGO health facilities respectively, the formal health care system in Uganda only reaches 54% of the children that require prompt and appropriate treatment, an estimate still below the 60% Abuja target [18]. In addition, the high prevalence of perceived treatment failure (38%–56%) in the alternative sources of treatment action necessitates a paradigm shift in the approaches aiming at improving treatment outcome. Countries such as Uganda have to increase the access to formal health care or HBMF because these two sources have the lowest perceived treatment failure (15%–23%). However, innovative ways are needed to improve treatment outcome in the non premium private sector. In Kenya, training shop-keepers improved correct treatment with chloroquine to approximately 65% [19]. More recently, in Uganda better performance was observed after negotiating with the informal drug shops, [20] and in Tanzania accredited drug-dispensing outlets (ADDOs) that supply antimalarial medicines recommended by the national policy perform better than those that are not accredited. Presently, the international community is lobbying for globally subsidized ACTs. However, there is a need to go beyond subsidies, because ready availability of ACTs without the supporting activities such as training, guidelines, job aids, group processes, regular supervision and monitoring will not achieve optimal results [21,22].

Presence of vomiting, convulsions and illness in the previous month were all independently associated with a high perceived treatment failure, suggesting that the CDDs need to look for these signs and symptoms and promptly refer those children they can not manage. In Ghana, community based agents (CBAs) who were provided limited skills to establish through further enquiry and observation the overt signs that could indicate severe disease performed better than those who had not [23].

As expected, children who were started on treatment after 24 hours of fever onset were 10 times more likely to experience treatment failure compared to those who started treatment within 24 hours of fever onset. Similarly, those taking HOMAPAK over the recommended three days duration were three-times less likely to experience treatment failure. These findings highlight the negative impact of non-compliance with national and international guidelines and suggest that the HBMF strategy needs a sustained communication strategy that addresses client and provider adherence to guidelines.

Diarrhea, vomiting, cough, failure to feed, convulsions and headache were the most common symptoms accompanying fever among the children. However, many symptoms for uncomplicated malaria overlap with those of severe malaria and acute respiratory infections (ARI) [24,25]. HBMF is intended for uncomplicated malaria, but in some cases severe malaria and patients with ARI are sometimes treated with HOMAPAK. This is the most likely explanation for the lower perceived treatment failure in the formal health care compared to HBMF because other causes of fever could have been investigated in the formal health care, improving the treatment outcome.

There are some limitations in this study. First, the caregivers could have given information they thought was desirable and acceptable or they could have exaggerated information in anticipation of assistance from the researchers. If this was the case, then the perceived treatment failure that was observed is likely to be biased on the favorable side, implying that our conclusions are really modest. Second, recall bias could have occurred. Minimizing recall bias was attempted through restricting interviews to the fever episodes that occurred two weeks preceding the study. Third, measurement of time from fever onset to treatment action is likely to be inaccurate because in rural areas people often tell time based on the position of the sun or from the occurrence of major events. Harmonizing the time reported by caregivers and the actual time to treatment action may have been biased leading to over or under estimation of time to treatment action. However, categorizing time into days reduces such inaccuracies. Finally, there could have been differences between research assistants in the measurement of the different study items (Inter-observer variability). Regular meetings between the research assistants and the investigators were held in an effort to reduce inter observer errors.

The caregivers perceived treatment failure in HBMF was almost 25%, suggesting that the present regimen has sub-optimal treatment efficacy. Implementation studies are urgently needed for the rational deployment of a more effective regimen in HBMF. In addition, Uganda should develop an information, education and communication (IEC) strategy that will improve adherence to HBMF guidelines. Further, there is a need to setup easy-to-reach and well facilitated formal health units in areas served by HBMF to quickly manage severe cases so as to limit the temptation to solely rely on HBMF to treat severely ill children. Finally, to ensure continuity of HBMF, especially in rural areas where the available non-formal alternatives for fever management are not as effective as HBMF, sustainable ways of facilitating the CDDs should be explored.

## Conflict of interest

The author(s) declare that they have no competing interests.

## Authors' contributions

**MM **conceived the objectives, developed the study design, supervised data collection, analyzed the data and participated in manuscript writing.

**EM **moderated the focus group discussions, participated in the development of survey tools and supervised data collection

**FK **contributed to the study design, participated in developing the data analysis plan and participated in manuscript writing.

**CK **contributed to the study design, participated in developing the data analysis plan and participated in manuscript writing.

**AOT **contributed to the development of the concept, advised on the study design, participated in developing the survey tools and data analysis plan and was lead person in writing the manuscript.

All authors read and approved the final manuscript.
